# Exploratory Studies on the Potential Health Benefits of Spices and Herbs

**DOI:** 10.1093/nutrit/nuaf247

**Published:** 2026-05-26

**Authors:** Guy H Johnson

**Affiliations:** McCormick Science Institute, Hunt Valley, MD 21031, United States; Department of Food Science and Human Nutrition, University of Illinois at Urbana-Champaign, Urbana, IL 61801, United States

**Keywords:** bioavailability, body composition, diet, fruits & vegetables, health, inflammation, physiology

## Abstract

This review is an analysis of reported studies that address the broad categories of diet quality and metabolic health. Among the noteworthy findings was that offering vegetables with a reduced-fat dip containing herb and spice flavors increased tasting, liking, acceptance, and/or consumption of vegetables among preschool children 3-5 years old. Consumers reported increased liking of an herb and spice–modified low-salt (LS) soup after repeated exposure, while no such increase was seen for its LS counterpart without spices and herbs. A study among cafeteria patrons reported similar appreciation for a LS legume-based mezze with herbs and spices and its counterpart with 50% higher salt, suggesting that the addition of herbs and spices is a feasible strategy to reduce salt content while maintaining consumer acceptance. Studies in the category of metabolic health have reported that speed of working memory was increased by an acute low dose of rosemary (750 mg) provided in tomato juice among free-living elderly study participants (65–90 years of age), whereas the opposite effect resulted from the highest dose (6000 mg). An ex vivo study among young adults (20–39 years of age) found that consumption of paprika, rosemary, ginger, heat-treated turmeric, sage and cumin for 7 days protected peripheral blood mononuclear cells (PBMCs) from DNA strand breaks while black pepper, cayenne pepper, cinnamon, clove, oregano (heat-treated or dried), and turmeric (no heat treatment) did not. A study among overweight/obese (mean BMI = 27 kg/m^2^) women provided with dietary red pepper and turmeric for 4 weeks demonstrated that these spices did not affect oxidative stress or inflammatory markers compared to a placebo, and another study showed that black pepper did not affect 24-hour energy expenditure or substrate utilization among postmenopausal women when provided at 0.5 mg/meal.

## STUDIES ON DIET QUALITY

### Vegetable Liking and Consumption Among Preschool Children

Savage et al.^1^ used a within-subject protocol to evaluate the effects of herb/spice-flavored, reduced-fat dips on willingness to taste, liking of, and consumption of vegetables by pre-school children. Participants were a convenience sample of children ranging in age from 3 to 5 years who attended a local child-care center in central Pennsylvania. Vegetable consumption among preschool students does not meet recommended intakes, and a considerable number of students consume no vegetables during a typical day.^2^ The rationale for this study was to determine if such consumption could be increased by providing vegetables in conjunction with an unflavored reduced-fat dip and a reduced-fat dip flavored with herbs.

The study protocol was conducted over a 6-week period. First, the familiarity of the children with and liking of 6 common vegetables and 5 dips (reduced-fat plain, herb, garlic, pizza, and ranch) were assessed to identify a vegetable that was liked, a vegetable that was disliked, and a dip that was liked by each participant. Two experiments were then conducted to evaluate the effects of a reduced-fat dip (plain or herb) on preschool children’s willingness to taste and liking of vegetables (Experiment 1), and the effects of serving vegetables with and without an herb-flavored dip on their consumption of vegetables (Experiment 2). Each experiment lasted 2 weeks; both were within-subject experiments, with children receiving all experimental conditions. Children in both experiments visited the tasting station 1 at a time in a room apart from their classmates.

#### Experiment 1

Each child participated in 2 short tasting sessions, tasting 3 vegetables (1 liked, 1 disliked, and 1 refused) with a preferred herb dip and plain reduced-fat dip as determined in the familiarity and liking protocol. Half of the children were given the plain dip and half their favorite herb-flavored dip in the first tasting session, and the order was reversed for the second session, with a 10- to 15-minute break between. Each child was told the names of the vegetables given, encouraged to taste the liked vegetable/dip combination first, and then allowed to choose which vegetable to taste next with the dip. After tasting the combination, the child rated it as “yummy,” “just okay,” or “yucky.”

#### Experiment 2

Each child participated in 4 snack sessions, administered on 4 different days, to assess the impact of dip availability conditions (with or without the child’s preferred pizza or ranch dip) on *ad libitum* intake of 2 vegetables. Yellow squash and celery were chosen because parents had indicated that their children were unfamiliar with these vegetables at study entry, and because these were the most disliked vegetables in the familiarity and liking session. The amount of each food item consumed (in grams) was determined by subtracting the post-snack weight from the pre-snack weight of the food.

#### Experimental Dips

The reduced-fat plain dip was formulated from Miracle Whip (Kraft Foods Inc) light sour cream and 2% milk. The reduced-fat herb dips were formulated from the plain dip with added herbs and/or spices and were labeled “pizza” or “ranch.” For the pizza dip, the following herbs and spices were added to the plain dip: basil, oregano, parsley flakes, onion powder, garlic powder, black pepper, Romano cheese powder, cheddar cheese powder, and paprika. For the ranch dip, the following herbs and seasoning were added to the plain dip: onion flakes, parsley flakes, dill, and salt. The 43-g (3.5-tbsp) servings of both the plain and herb dips provided 50 kcal, had a low energy density of 1.16 kcal/g, and contained 4 g fat (of which 1.5 g was saturated fat) and 90 mg sodium.

#### Results

Thirty-four of 46 participants completed Experiment 1. Compared with the vegetable alone, significantly more participants indicated that they liked the vegetable with plain dip (*P *< .001) and also liked the vegetable with herb dip (*P *< .001). There was a preference for the herb dip compared with the plain dip (*P *< .01). The children were more than twice as likely to reject the vegetable alone, rather than the vegetable paired with the plain dip (odds ratio [OR], 2.21; 95% CI, 1.08-4.53; *P *= .03); they were 3 times more likely to refuse to eat the vegetable alone compared with eating the vegetable paired with the herb dip (OR, 3.43; 95% CI, 1.60-7.33; *P *= .001). Rejection of the vegetable with dip did not differ by type of dip. Children ate more celery with dip than celery alone (*P *< .05) and also ate more squash with dip than squash alone (*P *< .05). The quantity of celery eaten increased 62% when it was paired with the herb dip; the quantity of squash eaten increased more than 2-fold when it was paired with the herb dip. The type of dip (pizza vs ranch) did not affect the amount of vegetable consumed.

The authors concluded, “Preschoolers offered vegetables paired with their preferred reduced-fat herb dip (pizza or ranch) were more willing to try vegetables and were less likely to reject them, compared with when they were served the vegetable alone or with a plain reduced-fat dip. Furthermore, they ate more of previously disliked vegetables when the vegetables were paired with their preferred herb dip…For parents, child-care providers, and staff at child-care facilities and schools, pairing a vegetable with a liked herb dip is a simple but useful strategy for increasing children’s willingness to taste, liking of, and consumption of vegetables.” A limitation of the study is that Experiment 2 did not include a treatment in which children tasted celery and yellow squash with the plain reduced-fat dip. The inclusion of such a treatment, “…would have provided a more definitive answer to the question of whether the children’s preferred reduced fat herb dip was mainly responsible for their increased vegetable intake, or whether a dip of any liked and familiar flavor might achieve the same improvement in vegetable intake.” Nevertheless, the study provides insights about a strategy to increase vegetable intake in this population.

### Liking of Lower-Salt Tomato Soup

Sodium chloride contributes texture and salty taste while decreasing bitterness and enhancing desirable flavors in may processed foods.^3^ However, dietary intakes of sodium are much higher than the nutritional requirement and are associated with increased risk of hypertension (and ultimately coronary heart disease).^4^ The use of spices and herbs to flavor foods with reduced salt is a preferred method because it results in a clean label and avoids the declaration of unfamiliar “chemical” components. Therefore, Ghawi et al.^5^ studied the effects of herb and spice blends used to enhance consumer acceptability of a LS tomato soup (0.26% w/w). Study participants (*n* = 148) scored their liking of tomato soup samples over 5 consecutive days. The first and last days were pre-and postexposure visits during which all participants rated 3 tomato soup samples, standard, low salt, and low salt with added herbs and spices. The middle 3 days were the repeated exposure phase during which participants were divided into 3 balanced groups, those consuming the standard soup, the LS soup, or the LS soup with added herbs and spices.

#### Soup Formulations

Three different herb and spice combinations added to the LS soup were tested for acceptance at the beginning of the study (Visit 1) in order to determine the most liked variant to progress to the main study: A basil seasoning modification that included basil, black pepper, celery, and garlic; a cumin/coriander modification formulated with cumin, coriander, ground celery seed, and garlic; and an oregano modification with added oregano, bay leaves, garlic, celery, and black pepper. No differences were found between these formulations in the liking of flavor, texture, appearance, and aftertaste, nor in the familiarity and the flavor intensity. However, hierarchical cluster analysis showed the oregano modification had the highest mean liking score for any 1 particular cluster, did not have a liking score as low as the basil modification in any particular cluster, and across the whole group had a higher rating for familiarity than the cumin and coriander modification (although this finding was not significant). On the basis of these findings it was decided to progress the oregano modification to the repeated exposure study.

#### Results

In the pre-exposure visit (Visit 2), consumer mean ratings of overall liking and liking of ,flavor were significantly higher for the standard soup (*P* < .0001 for both) and no significant difference was found between the oregano modification and the LS control soups. In the postexposure visit (Visit 6), consumer ratings had the same trend as those in the pre-exposure visit with regard to the overall liking, liking of flavor, and flavor intensity. In addition, at Visit 6 the consumers were asked to rate their perception of the salty, sour, and sweet tastes of the soups. Importantly, consumers found the oregano modification soup to be significantly saltier than the LS control, despite matched sodium contents (*P* = .017). However, these consumers did not find the LS control to be significantly less salty than the standard soup.

**Figure 1.**
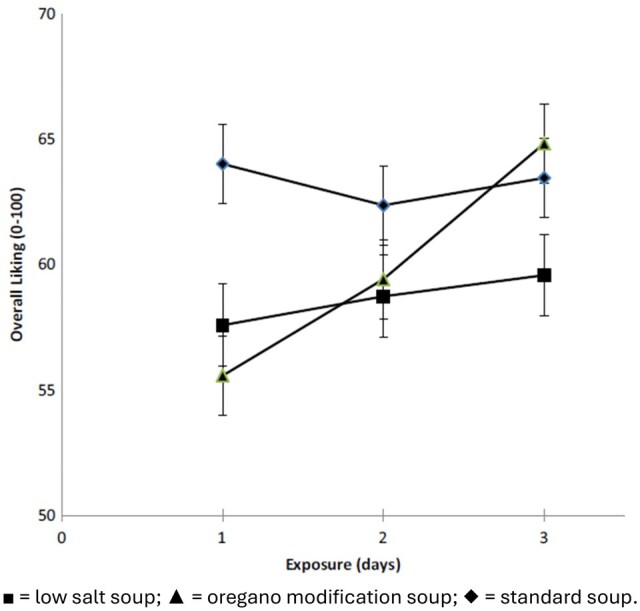
Overall Liking Development of Tomato Soups Across the Repeated Exposure Days

Each group of participants was exposed to a full portion of the same tomato soup for 3 consecutive days (Visits 3-5). Different trends were observed during the exposure course for each group. Over the exposure period the overall liking of the standard soup and the LS soup remained stable whereas the liking of the oregano modification soup increased over the 3-day exposure as shown in Figure 1 (t[285] = 2.34; *P* = .02).

The authors concluded that reducing salt in tomato soup led to an immediate decline in consumer liking and that incorporating herbs and adding spices and herbs did not cause an instant enhancement of liking. However, the herb and spice blends used in this study did enhance the perception of salty taste and compensated for a 53% reduction in added salt. In agreement with the psychological literature, in this study we found that initial liking was not static but developed over repeated exposure. Repeated exposure to the reduced-salt tomato soup modified with the addition of oregano, bay leaves, garlic, and black pepper led to a significant enhancement in the overall liking and liking of flavor, texture, and aftertaste, whereas no changes were observed for the standard and LS tomato soups during the repeated exposure phase (see Figure 1). Although there was a positive trend in increasing the liking of the developed tomato soup between pre- and postexposure, where it was compared directly with the standard salt soup, this trend was not statistically significant. Additional studies will be needed to determine whether such a change postexposure would be significant following a longer exposure period or across a larger consumer group. In addition, the current study provided the soup unaccompanied as a main course with no bread, which is not common practice in the United Kingdom. Studies with soup provided in a more conventional setting may be enlightening. Nevertheless, the current findings should encourage manufacturers to reduce salt in certain food products and increase the addition of carefully selected herbs and spices.

### Liking and Consumption of Lower-Salt Legume-Based Mezzes

As noted above, sodium chloride is an important component of foods from a sensory perspective, but dietary intakes greatly exceed recommended amounts, and sodium is associated with increased risk of hypertension, which is predictive of cardiovascular disease.^3^^,^^4^ Legumes are low in fat and densely packed with proteins, complex carbohydrates, fiber, and B-vitamins, and they are rich in several micronutrients, such as folate and iron.^6^ Legume consumpion is also associated with reduced risk of type-2 diabetes, cardiovascular disease, hypertension, obesity, and cancer.^7–9^ Therefore, Dougkas et al.^10^ studied the effects of adding spices and herbs to standard and reduced-salt legume-based mezzes on the liking, food intake, and appetite ratings among adults living in France. The experiment was conducted in 3 phases. Phase I was devoted to the development and determination of the most liked herb and spice modifications for use in further study and confirmation of differences in perception of saltiness and spiciness. Phase II consisted of evaluation of the overall liking of the different versions of the selected recipe in meal sessions (1 week apart), as a measure of absolute liking, and Phase III included similar assessments in which all 4 versions of the legume-based dish were evaluated during the same session, as a measure of relative liking.

#### Mezze Formulation

In the study by Dougkas et al*.,*^10^ the base mezze consisted of cooked chickpeas, cooked red lentils, olive oil, tahini, lemon juice, and water. Four different spice/herb combinations using this base were subject to consumer testing in Phase I:

- Curcumin blend modification: curcumin (0.8 g), ginger (0.4 g), shallot (0.4 g), and garlic (0.4 g)
- Ginger modification: ginger (1 g)
- Paprika blend modification: paprika (0.6 g), tomato (0.6 g), coriander (0.4 g), and garlic (0.4 g)
- Cumin blend modification: cumin (0.4 g), shallots (0.6 g), garlic (0.4 g), and spinach coulis (10 g)

#### Results

**Figure 2.**
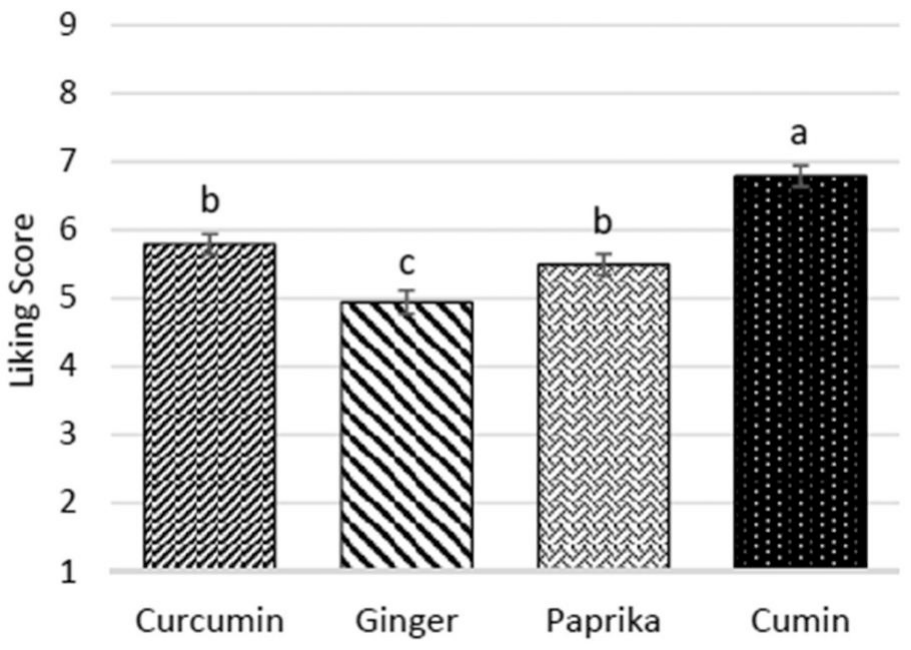
Liking of Herb- and Spice-Containing Mezzes

The least and the most appreciated mezzes determined by consumer testing in Phase I were the ginger (*P *< .05) and the cumin blend modification (*P *< .001), respectively. There was no significant difference between the curcumin and paprika blend modifications (*P *= .325), as shown in Figure 2.

Four versions of the cumin-containing mezze were subjected to further testing at the living Lab restaurant (real but controlled environment) of the Research Center of the Institut Paul Bocuse in Écully, France: A standard version with 0.8% salt (S), a lower salt formulation with 0.4% salt (LS), and both versions formulated with spices and herbs (SHS and LSHS, respectively).

**Figure 3.**
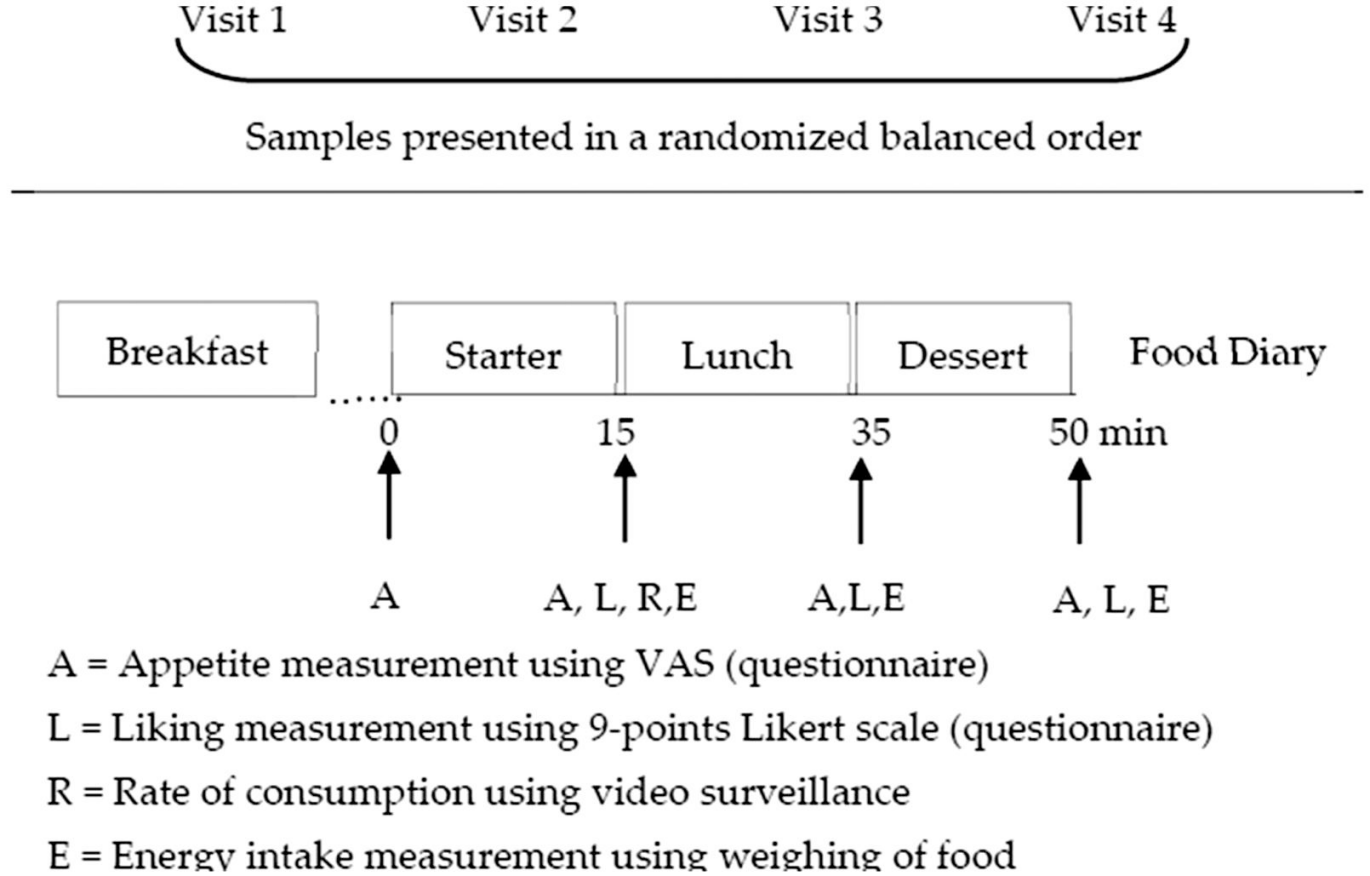
Schematic Representation of the Experimental Procedure

Participants were asked to visit the test site on 5 occasions, with at least a 1-week interval between visits. The first visit was a familiarization visit to the environment and experimental procedures, questionnaires, and test foods. Data recorded during this visit were not included in the results. Participants arrived at 12:00 pm and left around 1:30 am, a time period representing a typical French lunchtime. A food diary was provided and filled out by consumers during the study days. On arrival at 12:00 pm., the study participants sat down with their relatives, colleagues, or friends to simulate consumption in a real ecological context. The study participants answered a few questions that allowed a check on compliance with the instructions of having the same dinner and breakfast and refraining from physical activity and smoking. Appetite ratings were assessed before the mezze, which was served as a starter and at specific time intervals throughout the study after the starter, the main dish, and dessert were provided.^10^ The experimental day procedure is represented schematically in Figure 3.

**Figure 4.**
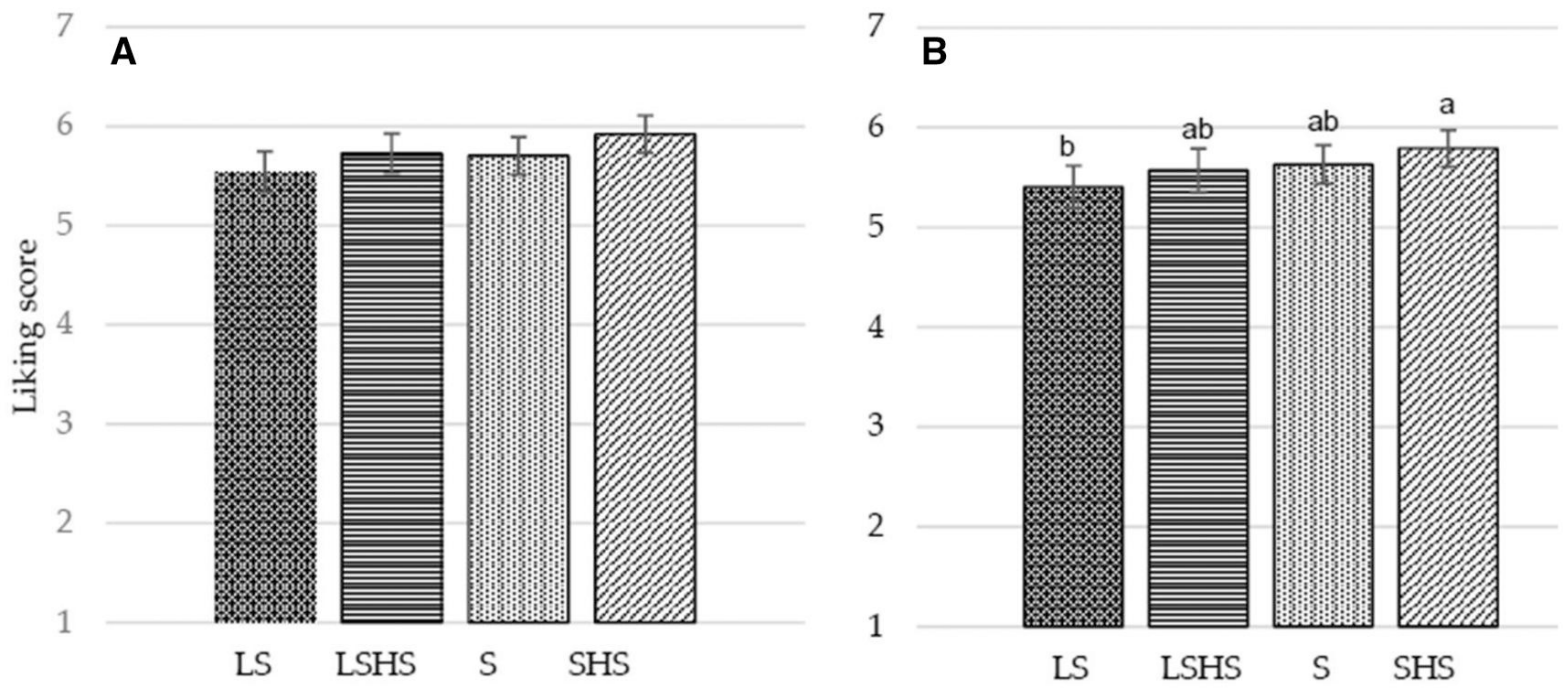
Liking and Flavor Liking Scores for Legume-Based Mezzes When Presented Separately 1 Week Apart

Ratings for liking overall and liking of taste obtained in the cross-over study (*n* = 94) of the 4 different versions of the cumin blend legume-based mezze, with each version being tasted in a different session, are summarized in Figure  4A and B.

There was a test product effect for the liking of taste (*P *= .044) (Figure 4B). The taste score was higher for the SHS compared with the LS mezze (*P *= .027), whereas there were no differences between those and the S and LSHS versions (*P *> .05). A similar trend was observed for the effect of the mezze on overall liking, yet without reaching significance (*P *= .065) (Figure 4A).

**Figure 5.**
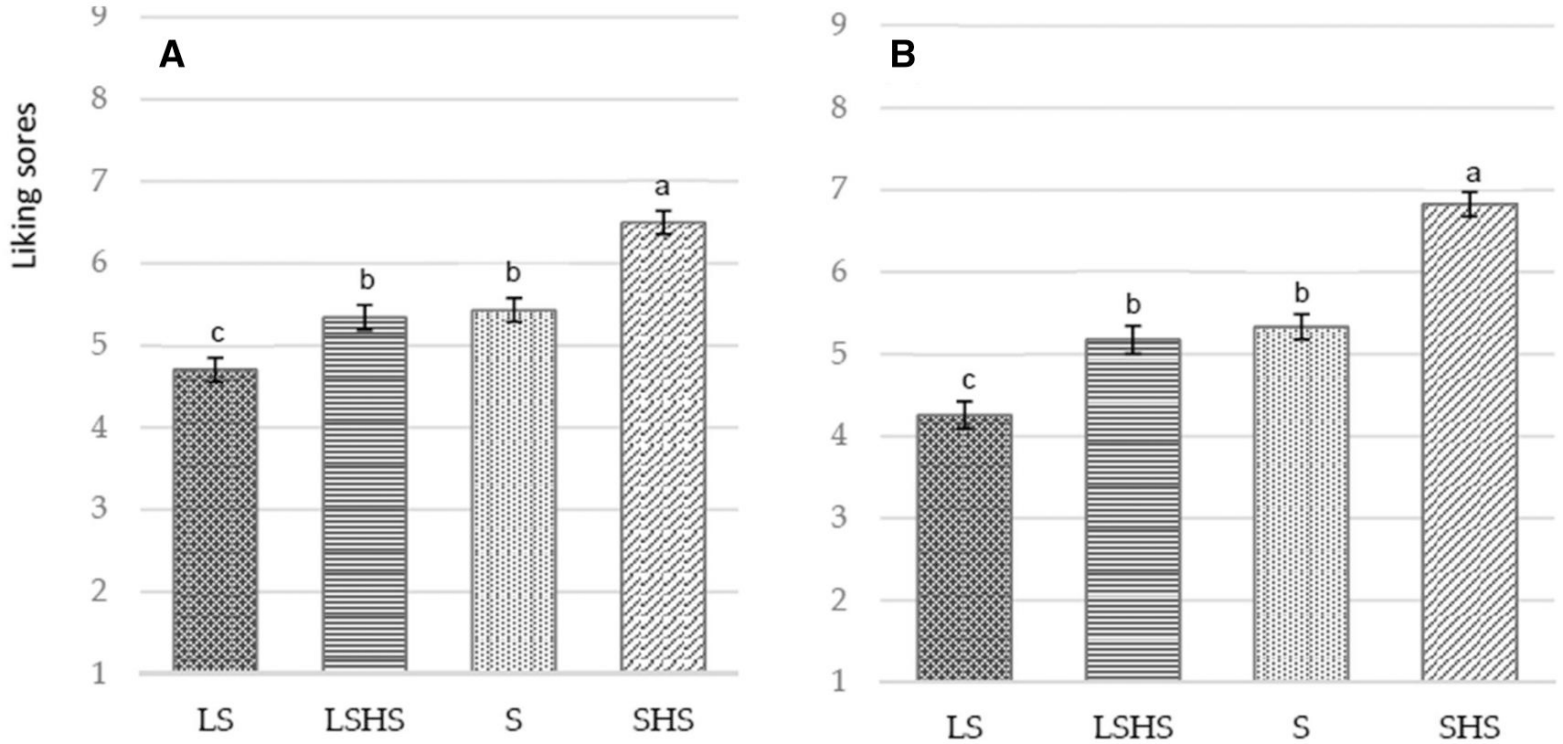
Liking and Flavor Liking Scores for Legume-Based Mezzes When Presented at the Same Session

Liking scores for the test mezzes when presented at the same session are presented in Figure  5A and B.

The mean ± SD values for the least appreciated version for overall and taste liking were 4.70 ± 0.15 and 4.26 ± 0.16, respectively, for the LS and for the most appreciated version were 6.50 ± 0.14 and 6.83 ± 0.15, respectively, for the SHS. Significant differences were observed between the standard-salt and LS mezzes, independent of the presence of herbs and spices (*P *< .001). However, there was no significant difference between S and LSHS versions, either for overall liking (*P *= .941) (Figure 5A) or for taste appreciation (*P *= .830) (Figure 5B). According to the analysis on the level of salt and herbs and spices (2-factor analysis), the effect of herbs and spices was as large as the effect of salt on overall liking (*F*[1, 360] = 56.3, *P *< .001; *F*(1, 360) = 69.0, *P *< .001, respectively). However, the effect of salt (*F*[1, 360] = 108.8, *P *< .001) was larger than the effect of herbs and spices on liking of taste (*F*[1, 360] = 85.2, *P *< .001).

The authors noted that a strength of the study was that it was conducted in an ecological environment representing a usual out-of-home environment. However, the lack of controlled experimental conditions for the sensory evaluation and presence of other people may have influenced the results. In addition, the findings are not generalizable to other populations or cultures with preferences for different spices and frequencies of legume consumption. The fact that the test legume was provided as a starter portion of the meal (typical of French restaurants) leaves open the possibility that consumption was impacted by expectations of subsequent parts of the meal. Further studies are needed to address these questions.

The authors concluded, “Overall appreciation and taste scores were similar between the LS with added herbs and spices and the standard-salt legume-based mezze, without differences in food consumption. These findings suggest that incorporating herbs and spices into reduced-salt food items is a feasible strategy for achieving a 50% reduction in salt content without sacrificing the hedonic liking of LS legume-based mezzes in a real restaurant setting.”^10^

## STUDIES ON PHYSIOLOGICAL EFFECTS

### Rosemary and Cognitive Function

Pengelly et al.^11^ studied the acute effects of dried rosemary (*Rosmarinus officinalis*) on cognitive function among 8 male and 20 female nonsmoking volunteers between 65 and 90 year old (mean age, 75 years). The study participants were in a stable state of health with no confounding medications and able to complete a computerized battery of cognitive tests on laptop computers via local media and networking. A randomized, placebo-controlled, double-blinded, repeated-measures, crossover protocol was used to administer the Cognitive Drug Research (CDR) test battery.^12^

#### Procedure

Powdered rosemary was added to a commercial reduced-sodium tomato juice (Campbell’s [Camden, NJ, United States]). On each study day the study participants received a single 16-ounce (458 mL) drink of this beverage containing 1 of the following doses of rosemary: (1) no rosemary; (2) 750 mg dried rosemary; (3) 1500 mg dried rosemary; (4) 3000 mg dried rosemary; or (5) 6000 mg dried rosemary. Doses of rosemary higher than would typically be consumed in a culinary setting were included due to the possibility that a that a large dose would be necessary to exert an effect after a single exposure in this acute study. Additionally, crude rosemary powder was used in a dietary formulation rather than a pharmaceutical extract to retain the pharmacokinetic profile of ordinary culinary consumption. Each dose was co-administered with colored methylcellulose-filled capsules to confound distinction between the treated and untreated tomato juice. Study participants were informed that these capsules could be part of the treatment. Masking was achieved by the use of opaque containers with black drinking straws and by chilling the drink. A placebo consisted of the tomato juice without rosemary as described above.

The tests were performed under experimenter supervision on 5 separate 1-day treatment sessions every week for 5 weeks following a practice day. The order of intervention on the 5 study visits was determined by random allocation. Each study day comprised 5 identical testing sessions: a pre-dose testing session to establish baseline performance for that day, followed immediately by the allocated intervention and assessments at 1, 2.5, 4, and 6 hours following consumption. Participants were asked to refrain from alcohol at least 12 hours prior to assessments on these days.^11^

#### Cognitive Measures

A battery of tasks from the CDR System was administered. Parallel forms of the tasks were performed at different sessions to reduce practice effect on repeated assessment. The information in all tasks was presented on the screen of a notebook computer, and with the exception of the written word recall tasks, the responses were recorded via a response module containing “NO” and “YES” buttons. The battery took about 25 minutes to perform.

The individual task outcomes from the battery were collapsed into 5 cognitive “factors,” as recommended by the CDR. Two of these factors concern attention: “power of attention” (sometimes called “speed of attention”), which reflects the ability to focus attention, whereas continuity of attention (or “accuracy of attention”) reflects the ability to sustain attention.

**Figure 6.**
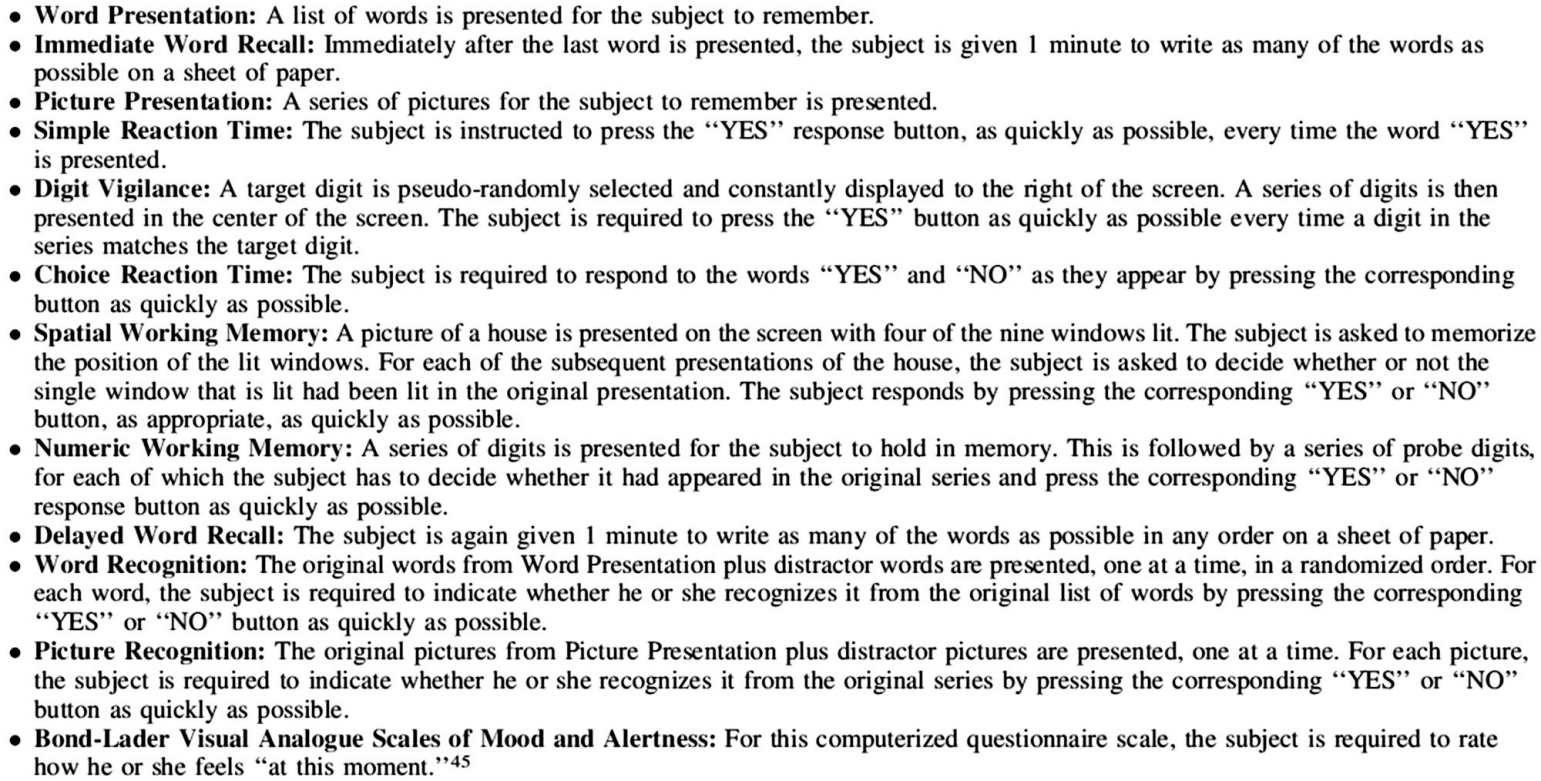
Cognitive Drug Research Tests

“Quality of working memory” reflects the ability to successfully hold numeric and spatial information temporarily in working memory, whereas “quality of episodic memory” reflects the ability to store, hold, and subsequently retrieve verbal and non-verbal information in long-term (episodic) memory. “Speed of memory” reflects the time taken to successfully retrieve information from both working and episodic memory.^11^ More detailed information about these tests is presented in Figure 6

#### Results

The results of the study are summarized in Table 1. There was a main effect of treatment for “speed of memory” measures (F4,96 = 7.19, *P *< .0001) that was dose specific. At 750 mg there was a significant improvement (*P *= .01), and at 6000 mg there was a significant impairment (*P *< .01) compared with placebo. With all treatments including placebo the participants showed a significant impairment compared with baseline except with the 750-mg dose, which showed negligible difference from baseline. “Continuity of attention” was significantly impaired at 1500 (*P *< .001), 3000 (*P *= .04), and 6000 mg (*P *< .001) doses, and “quality of working memory” was significantly impaired compared with placebo at 750 (*P *= .02), 1500 (*P *= .01) and 6000 mg (*P *= .01). However, the differences are much smaller when compared with baseline. There were no effects for the “power of attention” and “quality of episodic secondary memory” scores.

**Table 1.** Composite Scores for Effects of Rosemary Powder on 5 Cognitive Factors^a^^,^^b^

Cognitive factor	Dose (mg)	**Composite score** ^c^	SE	*P* value
Power of attention, ms	750	−18.493	10.650	.085
	1500	3.818	10.225	.709
	3000	6.902	10.917	.528
	6000	3.657	10.534	.729
Continuity of attention, score	750	−1.016	0.643	.117
	1500	−2.230	0.618	<.001
	3000	−1.376	0.658	.039
	6000	−2.363	0.637	<.001
Speed of memory, ms	750	−231.920	91 204	.012
	1500	−96.826	87.592	.271
	3000	29.130	93.453	.755
	6000	253.830	91.390	.006
Quality of working memory (score)	750	−.157	0.065	.018
	1500	−.167	0.063	.009
	3000	−.098	0.067	.144
	6000	−.169	0.065	.011
Quality of episodic memory (score)	750	1.998	6.271	.750
	1500	−9.140	5.992	.131
	3000	−8.567	6.240	.174
	6000	−1.695	5.928	.775

a Source: Pengelly, et al.^11^

b All scores are differed from placebo.

c Means averaged across time points are presented with SEs and *P* values associated with main effects of treatment.

The authors concluded that rosemary powder at the dose nearest normal culinary consumption demonstrated positive effects on speed of memory—a potentially useful predictor of cognitive function during aging. There were significant deleterious effects on other measures of cognitive performance, although these were less consistent. It was also noted that the short-term nature of this study did not allow the real-world impact of regular consumption of rosemary to be observed. Furthermore, it is not possible to determine whether such consumption would result in cumulative benefits or adaptation by the brain. The results point to the value of future studies on effects of low doses of rosemary on memory and cognition over the longer term.^11^

### Bioavailability of Spices and Herbs

Percival et al.^13^ studied the bioavailability of spices and herbs among healthy young adults. The purpose of the study was to determine if culinary amounts of selected herbs and spices induced specific physiological changes in humans suggesting that the herbs and spices have bioavailable components. The quantity that participants consumed was intended to reflect that usually consumed in food and was based on the level used in the Sensory Unit at McCormick for quality control. For example, in the Sensory Unit cinnamon is tested at 0.15% and turmeric at 0.025% of the consumed food. Based on the National Health and Nutrition Examination Survey (NHANES) data, total food consumption was estimated to be 1100 g/d. Therefore, the minimum dose calculated for research purposes was based on the percentage that would be used in testing against 1100 grams of food, or 1.7 g/d for cinnamon and 0.3 g/d for turmeric. These calculations assume that all food in the 1100 g/d is spiced at that level, which overestimates normal spice consumption but is practical for research purposes and should not be considered a pharmacological level. The bioavailability of each herb or spice was determined using the serum from participants before and after 7 days of consumption of a specific herb or spice as reflected by changes in 3 measures: appearance of antioxidant activity in the serum, the ability of the serum to protect PBMCs from oxidative stress, and the ability of the serum to alter cell cytokine responses to inflammatory stimulation as determined by ex vivo inflammatory suppression and occurrence of DNA strand breaks in PBMCs.

#### Procedure

Thirteen groups of 10-12 generally healthy men and women 20-39 years of age were assigned to 1 of the test spices/herbs. After an overnight fast, participants had their baseline blood drawn and began twice-daily consumption of capsules containing a particular herb or spice. After 7 days, participants returned for a final blood draw 1 hour after consumption of the last capsule(s).

The test spices and herbs and daily amount in grams/d were paprika (1.7), cayenne pepper (0.3), Saigon cinnamon (1.7), cumin (2.8), turmeric (0.3), Mediterranean oregano (1.1), black pepper (2.8), rosemary (2.8), Dalmatian sage (1.7), clove (0.3), and ginger (2.8).^13^

#### Results

The antioxidant capacities determined for the various herbs and spices (subjected to simulated digestion with 2 mmol/L HCl for 30 minutes at 37° C, followed by neutralization to pH 7.0 with sodium bicarbonate) were very different from each other and ranged from 162.8 µM Trolox equivalents (TE) per gram for black pepper to 1952.0 µg TE/g for rosemary after. Antioxidant activity was assayed in the serum collected from the participants 1 hour after final capsule consumption, but none was demonstrated. Even for rosemary, the herb with the highest antioxidant capacity, no significant increase in serum antioxidant activity was detected by either the DPPH or the ORAC assay.

Statistically significant data regarding protection against DNA strand breaks were obtained from 6 of the 13 herbs and spices. Depending on the herb or spice, significant differences were seen for either the number of strand breaks per cell or the percentage of the cell population with strand breaks; or for both. The specific herbs and spices that protected PBMCs from DNA strand breaks with or without induction with hydrogen peroxide were paprika, rosemary, ginger, heat-treated turmeric, sage, and cumin. Results from the other 7 treatment groups (black pepper, cayenne pepper, cinnamon, clove, oregano [heat-treated or dried], and turmeric [no heat treatment]) did not achieve significance.

**Figure 7.**
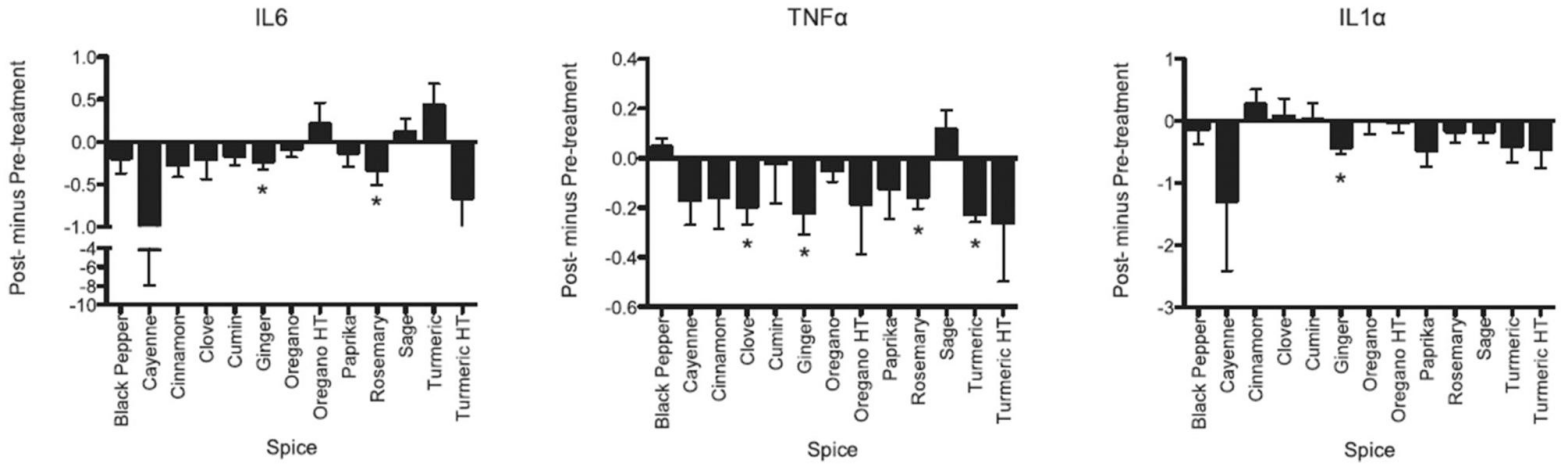
The Effects of Treating OxLDL-Challenged THP-1 Cells with Serum from Individuals Before and After Consumption of Herb or Spice Capsules

Serum from individual participants was added to media (10%, v/v) and used to treat Tohoku Hospital Pediatrics-1 (THP-1) cells that had been subjected to inflammatory stimulation with oxidized low-density lipoprotein (oxLDL) at a concentration determined from preliminary studies (50 lg/mL). As shown in Figure 7, the overall expression of inflammatory markers was lower when THP-1 cells were treated with serum from individuals after capsule ingestion compared to cells treated with serum collected prior to dietary intervention with the herbs and spices. Ex vivo expression of interleukin-6 (IL-6) was decreased by ginger and rosemary, whereas IL-1α mRNA expression was significantly decreased only by ginger. Tumor necrosis factor-α (TNF-α) proved to be the most sensitive marker of responsiveness to serum ex vivo, as sera from individuals consuming clove, ginger, rosemary, and turmeric significantly reduced TNF-α expression in oxLDL-stimulated THP-1 cells.

In summary, herbs and spices that protected PBMCs against DNA strand breaks were paprika, rosemary, ginger, heat-treated turmeric, sage, and cumin. Paprika also appeared to protect cells from normal apoptotic processes. Of the 3 cytokine mRNAs studied (TNF-α, IL-1α, and IL-6), TNF-α was the most sensitive responder to oxidized LDL-treated macrophages. Clove, ginger, rosemary, and turmeric consumption was associated with significantly reduce oxidized LDL-induced expression of TNF-α. Serum from those consuming ginger reduced all 3 inflammatory biomarkers.

Ginger, rosemary, and turmeric showed protective capacity by both oxidative protection and inflammation measures. The inability to protect against an ex vivo oxidative insult means that the compounds consumed were either not bioavailable or were not available in sufficient quantity to provide protection. This type of distinction, however, could not be determined from the data. Nevertheless, the authors concluded that DNA strand breaks and inflammatory biomarkers are a good functional measure of a the bioavailability of a food.^13^

### Red Pepper/Turmeric and Inflammatory and Oxidative Stress

Nieman et al.^14^ studied the effect of 1 g/d of red pepper (RP) or 2.8 g/d of turmeric (TM) on a variety of inflammatory and oxidative stress markers among overweight adult women (mean body mass index [BMI], ≥27 kg/m^2^; 40-75 years old; *n* = 98). The study hypothesis was that supplementation with RP or TM would reduce inflammation and oxidative stress and improve vascular function in free-living, overweight females with underlying chronic inflammation (CRP ≥ 2 mg/L). We used a crossover design under double-blinded, placebo-controlled conditions with culinary levels of RP (1 g/d) and TM (2.8 g/d) that would be acceptable to US adults.

#### Procedure

Participants were randomized to RP or TM groups, and ingested RP, TM, or placebo (PL) supplements daily for 4 weeks under double-blinded conditions with randomized crossover to the opposite condition (spice or PL) following a 2-week washout period. Body composition, blood pressure, augmentation index, and blood samples were taken from all participants pre- and post-supplementation after an overnight fast for each of the 4-week supplementation periods. The spices and PL (refined white rice flour) were contained in identical-looking blue gelatin capsules (2/d for RP or PL, 5/d for TM or PL).

#### Results

Supplementation with RP or TM over a 4-week period had no influence relative to PL on body weight, percentage body fat, systolic blood pressure, augmentation index, or serum glucose. There were also no differences in any of the inflammatory or oxidative stress markers examined as presented in Table 2. The authors concluded, “These negative findings should be considered within the context of the culinary doses used for 4 weeks, the biomarkers chosen for this study, and number of participants. Future research should emphasize higher doses, spice mixtures and blends of selected phytochemicals, and longer supplementation periods to determine if humans receive health benefits from spice ingestion.”^14^

**Table 2.** Influence of Red Pepper (*n* = 31) and Turmeric (*n* = 30) Supplementation on Inflammatory and Oxidative Stress Markers^a^^,^^b^

Variable	Red pepper	Placebo	Turmeric	Placebo	Interaction *P* values
Serum CRP, mg/L					
Pre-study	7.64 ± 0.82	9.48 ± 1.71	8.05 ± 1.33	7.44 ± 0.98	.091
Post-study (4 wk)	8.13 ± 1.00	7.37 ± 0.94	6.85 ± 1.00	6.33 ± 0.88	.948
Plasma IL-6, pg/mL					
Pre-study	2.96 ± 0.85	3.43 ± 0.96	3.19 ± 0.66	2.73 ± 0.48	.064
Post-study (4 wk)	4.14 ± 1.06	2.80 ± 0.96	2.21 ± 0.34	2.75 ± 0.53	.136
Plasma IL-8, pg/mL					
Pre-study	4.81 ± 0.40	5.14 ± 0.52	4.93 ± 0.51	4.85 ± 0.49	.177
Post-study (4 wk)	5.08 ± 0.63	4.44 ± 0.41	5.11 ± 0.49	4.43 ± 0.51	.220
Plasma IL-10, pg/mL					
Pre-study	3.92 ± 0.64	3.78 ± 0.50	4.24 ± 0.63	4.25 ± 0.83	.812
Post-study (4 wk)	3.60 ± 0.46	3.71 ± 0.54	3.48 ± 0.54	3.59 ± 0.87	.881
Plasma TNF-α, pg/mL					
Pre-study	6.08 ± 0.78	5.79 ± 0.52	6.97 ± 0.63	6.33 ± 0.61	.811
Post-study (4 wk)	5.82 ± 0.52	5.28 ± 0.49	6.41 ± 0.63	6.04 ± 0.50	.630
Plasma F_2_-isoprostanes, pg/mL					
Pre-study	95.1 ± 4.0	99.8 ± 4.3	88.8 ± 2.6	91.2 ± 3.3	.744
Post-study (4 wk)	96.8 ± 4.4	100 ± 4.3	90.5 ± 2.7	92.3 ± 3.5	.883
Plasma oxidized LDL, U/L					
Pre-study	50.5 ± 4.7	47.8 ± 3.7	48.8 ± 2.9	52.2 ± 3.2	.599
Post-study (4 wk)	50.9 ± 4.3	46.6 ± 4.3	44.3 ± 3.0	44.6 ± 3.2	.373

Abbreviations: CRP, C-reactive protein; IL, interleukin; LDL, low-density lipoprotein; TNF, tumor necrosis factor.

a Values are mean ± SD unless otherwise indicated.

b Source: Nieman, et al.^14^

### Black Pepper and Energy Expenditure

O’Connor et al.^15^ studied the effect of 0.5 mg black pepper (BP) per meal on energy expenditure (EE), respiratory quotient (RQ) and other parameters among 17 postmenopausal women (mean age 60.4 years) who spent 24 hours in a whole-room indirect calorimeter.

#### Procedure

In this studywe used a randomized, controlled, cross-over design with 1.5 g/d of BP vs a no pepper control (NPC). The participants consumed 60.8 g of low-sodium V8^®^ vegetable juice with or without 0.5 g BP at each of the 3 study day meals. This amount of BP was selected to be larger than the typical 0.3 g/d intake in this population, but still acceptable from a culinary perspective. A taste panel including 8 volunteers who rated 0.g, 0.7 g, and 1.0 g/serving of BP on a 5-point Likert scale deemed that a 3 times/d dose of 0.5 g/d was most acceptable. The participants reported to the unit following an overnight fast at 8 am and were sealed in the chamber and requested to remain seated or reclined, but awake, throughout the day. Breakfast (at 9 am), lunch (at 1:30  pm), and dinner (at 5 pm), were served through an air-lock passage. The V8^®^ with or without BP was served alongside each meal (consumed within 30 minutes). At 2 pm blood was sampled through an iris port. Study participants were requested to prepare for bed at 10 pm. At 10;30 pm, lights were turned off and participants were asked to lie down even if not sleeping. Study participants were woken up at 06:30 am. At 07:15 am, participants exited the chamber.

#### Results

Twenty-seven women were recruited. Following screening, 26 participants were deemed eligible. Twenty participants commenced the study, of whom 18 completed all study days. One participant was excluded due to poor compliance, leaving 17 participants in the final analysis. No differences were observed in 24-hour EE or components of EE, namely resting metabolic rate, seeping metabolic rate, diet-induced thermogenesis, or activity-induced EE between the NPC and the BP treatments. No significant between-treatment differences were observed in 24-hour RQ or post-prandial area under the curve for RQ 4 hours after breakfast, lunch, or dinner. Activity (%) was comparable between the BP and NPC study days. Postprandial concentrations of gut peptides (glucagon-like peptide-1 [GLP-1], peptide YY [PYY], gastric inhibitory peptide [GIP], and ghrelin) were also comparable between BP and NPC treatments. Additionally, insulin, C-peptide, leptin, adrenaline, noradrenaline, and dopamine were unaltered by BP consumption.

The authors concluded, “Investigating the role of bioactive compounds is complex. Study design decisions should encompass population of choice, body composition and energy balance. This study suggests that acute consumption of 1.5 g of BP is well tolerated and significantly increases circulating piperine. However, this dose does not influence 24-hour EE, RQ, or postprandial biomarkers of satiety in postmenopausal, overweight White women under eucaloric conditions. Nevertheless, it is possible that BP administration in the between-meal or fasted state, a higher daily dose, or synergy arising from a combination with other thermogenic ingredients may have greater utility. Considering the potential benefits of thermogenic ingredients in the fight against obesity, continued research is warranted to determine populations who may maximally benefit from these dietary bioactives.”

## CONCLUSIONS

The studies briefly summarized in this article add to the body of knowledge regarding the potential health benefits of culinary spices and herbs. These ingredients have the potential to improve public health by increasing the appeal, and ultimately the consumption, of a healthy diet and/or by imparting beneficial physiological effects. As would be expected, the results of such studies are mixed. Nevertheless, the body of evidence as a whole provides proof of concept support for such studies and suggests multiple opportunities for additional research. For example, spices/herbs (S/Hs) are fundamental to the characterization of food across cultures, and additional research is necessary to understand which S/Hs are most likely to improve diet quality and/or metabolic health in different populations. In addition, little is known about the use of S/Hs as strategies for personalized nutrition with respect to age, sex, genetic endowment, microbiome status and other considerations. The use of S/Hs as part of the “Food as Medicine” movement also has potential. Studies designed to assess the ability of S/Hs to increase the appeal and/or effectiveness of prescribed diets on health-related outcome measures are limited.

In conclusion, the studies reviewed in this article, as well as many of the other studies in this compendium, support the notion that additional research is warranted to expand our knowledge on the ability of S/H to contribute to public health. Longer-term clinical trials designed to determine the real-world value of chronic exposure to a variety of S/H among differing populations are needed to complement the available short-term studies. It is hoped that this compendium will stimulate interest in such research.
